# Coordination of dendritic inhibition through local disinhibitory circuits

**DOI:** 10.3389/fnsyn.2015.00005

**Published:** 2015-02-26

**Authors:** Ruggiero Francavilla, Xiao Luo, Elise Magnin, Leonid Tyan, Lisa Topolnik

**Affiliations:** Department of Biochemistry, Microbiology and Bio-informatics, Université Laval; Axis of Cellular and Molecular Neuroscience, IUSMQQuébec, PQ, Canada

**Keywords:** GABA, interneuron, synapse, VIP, disinhibition

## Abstract

It has been recognized for some time that different subtypes of cortical inhibitory interneurons innervate specific dendritic domains of principal cells and release GABA at particular times during behaviorally relevant network oscillations. However, the lack of basic information on how the activity of interneurons can be controlled by GABA released in particular behavioral states has hindered our understanding of the rules that govern the spatio-temporal organization and function of dendritic inhibition. Similar to principal cells, any given interneuron may receive several functionally distinct inhibitory inputs that target its specific subcellular domains. We recently found that local circuitry of the so-called interneuron-specific (IS) interneurons is responsible for dendritic inhibition of different subtypes of hippocampal interneurons with a great impact on cell output. Here, we will review the properties and the specificity of connections of IS interneurons in the CA1 hippocampus and neocortex, and discuss their possible role in the activity-dependent regulation of dendritic inhibition received by pyramidal neurons.

## Introduction

In neocortical and hippocampal networks, a large diversity of GABAergic inhibitory inputs converges onto the dendrites of glutamatergic principal cells. Many of them may overlap within the same dendritic domain but remain segregated temporally due to specific inhibitory mechanisms that evolved to control the activity of dendrite-targeting interneurons. The vasoactive intestinal polypeptide (VIP) and/or calretinin (CR) expressing interneurons have been consistently associated with cortical dendritic disinhibition. For example, in the CA1 hippocampal area, three types of the so-called interneuron-specific (IS) interneurons have been shown to make symmetric contacts with interneurons selectively (Acsády et al., 1996; Gulyás et al., 1996). Type 1 (IS1) cells express CR and have a soma located in the oriens/alveus (O/A), stratum pyramidale (PYR) or radiatum (RAD). Type 2 (IS2) interneurons express VIP but lack CR: they have a soma located between the RAD and lacunosum-moleculare (LM), a dendritic arbor restricted to LM and axonal projections in the RAD targeting cholecystokinin (CCK)/VIP coexpressing basket cells. Type 3 (IS3) interneurons coexpress CR and VIP and may also express enkephalins with a soma located at the PYR and RAD border and dendrites extending into LM (Blasco-Ibáñez et al., 1998). IS3 cells have been reported to contact preferentially somatostatin (SOM)- and metabotropic glutamate receptor 1a (mGluR1a)-positive oriens–lacunosum moleculare (OLM) cells that are responsible for distal dendritic inhibition of CA1 pyramidal neurons (Acsády et al., 1996). In a second example, the majority of VIP+ terminals in the somatosensory cortex are made onto SOM-/mGluR1a- and calbindin (CB)-expressing interneurons that provide dendritic inhibition to the layer II/III and layer V pyramidal cells (Dalezios et al., 2002; Staiger et al., 2004). However, the physiological properties, functional connectivity, recruitment during network activity, and role of IS interneurons in cortical computations remained until recently unknown.

In the past several years, advances in transgenic and optical technologies have converged to enable researchers to target and manipulate specific cell types within highly heterogeneous inhibitory circuits. Using VIP-GFP mice, it became possible to characterize the properties and connectivity of VIP+ interneurons in acute hippocampal slices (Chamberland et al., 2010; Tyan et al., 2014), while mice expressing Cre recombinase and channelrhodopsin (ChR) or halorhodopsin under the control of the VIP or CR promoters have been successfully used to manipulate VIP+ and CR+ interneurons in slices and in awake mice (Lee et al., 2013; Pfeffer et al., 2013; Pi et al., 2013; Tyan et al., 2014). Here, we summarize current knowledge about the properties, connectivity and function of VIP+ interneurons in the hippocampus and neocortex. In particular, we concentrate on hippocampal CA1 IS3 cells that control the level of dendritic inhibition received by CA1 pyramidal neurons. It is not our intention to discuss CR+ cells in cortical circuits, as they represent a highly heterogeneous population of interneurons and have been thoroughly discussed in a recent review (Cauli et al., 2014).

## Properties and connectivity of IS3 cells

### Morphological and neurochemical features

**Figure 1:**
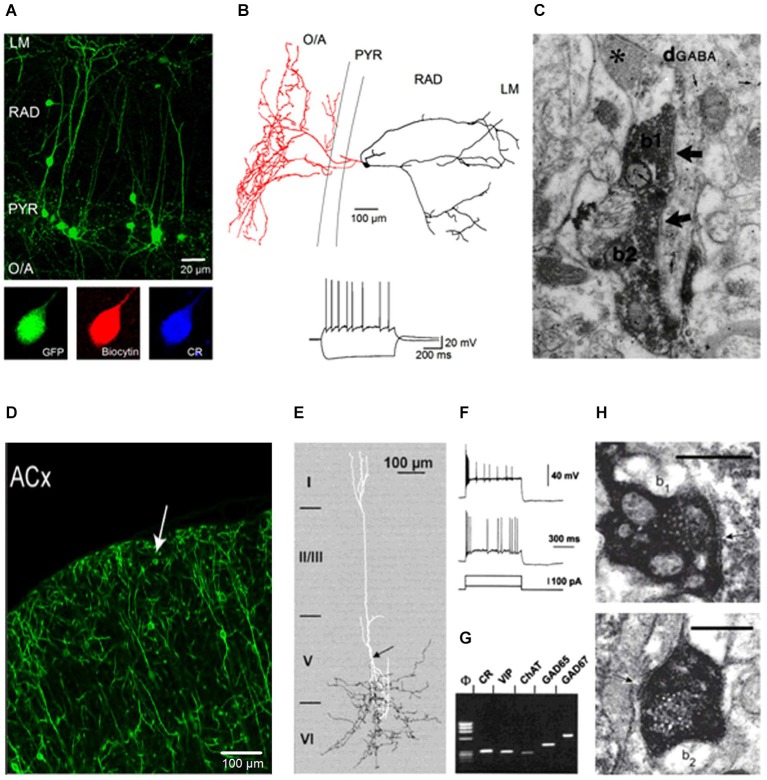
**Properties of IS3 interneurons in the hippocampus and CR/VIP co-expressing interneurons in the neocortex. (A)** Two-photon image (maximal projection of a *z*-stack) of the CA1 area from an acute hippocampal slice (300 µm) of a VIP-eGFP mouse showing the morphological features of VIP-positive interneurons in the CA1 area. Lower panel represents confocal images showing CR expression by IS3 interneurons in the CA1 area. **(B)** Anatomical reconstruction (the axon is shown in red, the dendrites are shown in black) of an IS3 cell that was recorded and filled with biocytin. Inset illustrates representative voltage responses of an IS3 interneuron to positive (50 pA) and negative (−100 pA) current injections (Modified from Tyan et al., 2014). **(C)** EM image showing VIP+ boutons of two neurons (b1 and b2) forming symmetrical synaptic contacts (arrows) on the same dendrite which is shown to be immunoreactive for GABA by the accumulation of gold particles (small arrows) (Data are from Acsády et al., 1996). **(D)** Confocal image of the auditory cortex (ACx) with the morphological features and layer distribution of VIP+ somata; arrow indicates a VIP+ interneuron in the first layer. Scale bar, 100 µm (Data are from Pi et al., 2013). **(E)** Anatomical reconstruction of a VIP+ interneuron with dendrites shown in white and axon shown in black. The arrow indicates the initiation point of a descending axon arborizing in the sixth cortical layer (Data are from Porter et al., 1998). **(F,G)** Electrophysiological and molecular properties of CR/VIP coexpressing interneurons in the neocortex. **(F)** Voltage responses to depolarizing pulses of 50 (lower trace) and 200 (upper trace) pA. The response consists of an initial burst followed by intermittent action potentials at an irregular frequency. **(G)** Single-cell RT-mPCR analysis showing the expression of ChAT, GAD65 and GAD67 mRNAs in CR/VIP coexpressing neocortical interneurons (Data are from Porter et al., 1998). **(H)** EM images of symmetric synapses (indicated by arrows) formed by VIP- presynaptic boutons (b_1_ and b_2_) with the soma of CB+ interneuron. Scale bars, 0.5 µm (Data are from Staiger et al., 2004).

The hippocampal CA1 IS3 cells have small round somata (13–18 µm) located in PYR or RAD with 2–3 primary dendrites of unipolar or bipolar orientation extending towards LM or LM and O/A, respectively (Figures 1A,B). In most cells, the primary dendrite extending to LM is particularly thick resembling that of pyramidal neurons (Figure 1A upper panel). Dendritic spines can be observed occasionally on proximal and distal branches. These cells send their axon primarily to the O/A but random collaterals can be found in PYR or RAD (Figure 1B upper panel). Accordingly, the major postsynaptic targets of IS3 cells reside in the O/A and correspond to O/A interneurons (Figure 1C). On the basis of immunohistochemistry, IS3 interneurons are defined as GABAergic cells that co-express the Ca^2+^-binding protein CR and neuropeptide VIP (Figure 1A lower panel) (Acsády et al., 1996; Freund and Buzsáki, 1996; Gulyás et al., 1996).

### Physiological properties

In acute hippocampal slices, IS3 cells have a resting membrane potential of −64 to −75 mV, suggesting that these cells are likely silent under basal conditions. However, compared with other interneuron subtypes, IS3 interneurons have a particularly high input resistance (400–600 MΩ) and a small rheobase (30–50 pA), which makes them one of the most excitable interneuron subtypes in the hippocampus. The properties of the action potential, including the spike threshold, the amplitude and the half-width are similar to those in other types of neurons (Tyan et al., 2014). Nevertheless, IS3 interneurons can distinguish themselves by a characteristic “irregularly spiking” firing pattern with an inter-spike interval varying broadly upon membrane depolarization (Figure 1B lower panel) (Chamberland et al., 2010).

### Connectivity

The IS3 axon shows extensive arborization within O/A with a cumulative axonal length of a single interneuron going up to 11 mm. Data from anatomical analysis (Acsády et al., 1996) and paired electrophysiological recordings (Tyan et al., 2014) showed that IS3 cells contact several distinct subtypes of O/A interneurons, including OLM, bistratified and basket cells as well as some other interneurons with somata, dendrites and axon located within stratum oriens [the so-called oriens–oriens cells]. The OLM cell is the preferential target of IS3 interneurons while the oriens–oriens and bistratified cells share most of the remaining IS3 inputs with a minor proportion of inputs made onto basket cells (Tyan et al., 2014). Taken together, these data indicate that the major role of IS3 interneurons is in coordinating the level of inhibition converging onto different dendritic domains of CA1 pyramidal neurons.

### Properties of IS3 synapses

The properties of IS3 synapses made on different targets have been examined using paired patch-clamp recordings (Tyan et al., 2014). In all dendrite-targeting interneurons, unitary inhibitory postsynaptic currents (uIPSCs) recorded at 0 mV at near-physiological temperature (32 ± 1°C) had a high failure rate of ~60%, small amplitude (10–25 pA) and varying kinetics (uIPSC rise time: 0.7–1.3 ms; uIPSC decay τ: 5–12 ms). The latter could result from the different dendritic location of IS3 synapses in distinct targets, although a target-specific GABA_A_ receptor composition cannot be excluded (Salesse et al., 2011). Variance-mean analysis has been performed at IS3–OLM synapses (Tyan et al., 2014). It revealed that an IS3 cell contacts an OLM through multiple release sites and produces uIPSCs with a quantal size of 5–6 pA. Repetitive firing of IS3 cells at 10–100 Hz does not result in any form of short-term plasticity at IS3–OLM synapses. However, efficient summation of slow uIPSCs occurring during 100-Hz firing of IS3 cells leads to a large inhibitory response in OLMs with a potential impact on their firing.

### Comparison with VIP+ interneurons in the neocortex

In neocortical regions, VIP+ interneurons have been classified as a sub-group of interneurons that express the 5-hydroxytryptamine 3a receptor (5HT3aR+), making up ~40% of 5HT3aR+ interneurons (Rudy et al., 2011). Similar to hippocampal IS3 interneurons, most neocortical VIP+ interneurons have a bipolar/ bitufted orientation with soma and dendrites located primarily in layers II/III or V (Figure 1D; Pi et al., 2013). These cells have dendrites located perpendicularly to the pial surface and branching within layers I and V. The axon of bipolar VIP+ interneurons originates from a primary dendrite and makes an extensive arborisation within layers V/VI (Figure 1E; Porter et al., 1998). A sub-population of neocortical VIP+ interneurons co-express CR (35% of VIP+ cells) and, therefore, may be similar to the hippocampal IS3 interneurons (Figure 1G; Kawaguchi and Kubota, 1997; Porter et al., 1998; Gonchar et al., 2008; Xu et al., 2010). It is to be noted that a fraction of neocortical VIP+ interneurons may also co-express CCK and, therefore, may correspond to VIP+ basket cells (Galarreta et al., 2004; Sugino et al., 2006).

Similar to hippocampal IS3 interneurons, neocortical CR+/VIP+ interneurons recorded in slices *in vitro* are hyperpolarized with a resting membrane potential of −62 to −74 mV (Porter et al., 1998). These cells have a high input resistance (240–2200 MΩ) and exhibit “irregularly spiking” firing pattern (Figure 1F; Cauli et al., 1997, 2000; Porter et al., 1998; Galarreta et al., 2004; Lee et al., 2010; Miyoshi et al., 2010). In support of their interneuron-selectivity, the results of ultrastructural, physiological and optogenetic analysis revealed that VIP+ interneurons prefer to contact several distinct subtypes of neocortical interneurons, including CB+, SOM+, VIP+ and parvalbumin-positive (PV+) cells (Figure 1H). In particular, electron microscopy studies have shown that VIP+ boutons onto PV+, CB+, SOM and VIP+ interneurons are homogeneously distributed across layers II to VI (Dalezios et al., 2002; Staiger et al., 2004; Dávid et al., 2007). Moreover, paired whole-cell recordings from neocortical layer II/III CR+/VIP+ interneurons showed that these cells prefer to contact several types of interneurons rather than pyramidal cells, including the multipolar CR+/VIP– cells (with a connectivity rate of 80%), fast spiking cells (30%), and PV+ multipolar bursting cells (27%) (Caputi et al., 2009). Furthermore, optogenetic studies using a VIP-Cre mouse model have shown that SOM+ interneurons represent the major target of VIP+ interneurons; in particular, the inhibition provided by VIP+ interneurons was much larger in SOM+ cells compared with PV+ interneurons in the visual and somatosensory cortices (Lee et al., 2013; Pfeffer et al., 2013). A similar observation was reported in the auditory and medial prefrontal areas (Pi et al., 2013), where activation of ChR2-expressing VIP+ interneurons elicited IPSCs primarily in SOM+ cells; albeit no difference in the amplitude of the ChR2-evoked IPSCs appeared between SOM+ and PV+ interneurons. In addition, optogenetic silencing of VIP+ interneurons strongly reduced the IPSCs recorded in neocortical SOM+ cells (Lee et al., 2013). Finally, CR+/VIP+ interneurons are coupled through gap junctions (with a connectivity rate of 63%) (Caputi et al., 2009), which may play an important role in synchronizing the activity of CR+/VIP+ interneurons with a great impact on the output of SOM+ interneurons. Taken together, these studies show that VIP+/CR+ IS interneurons are well positioned to modulate primarily the activity of local SOM+ circuits, providing dendritic disinhibition to cortical pyramidal neurons.

## Functional role of disinhibitory circuits

In the CA1 hippocampus, dendritic inhibition provided by the IS3 interneurons controls the firing rate and timing of OLM cells. The latter may be possible because of the dendritic initiation of the action potential in OLM interneurons (Martina et al., 2000). Furthermore, it has been shown that SOM+ dendrite-targeting OLM as well as bistratified cells may be responsible for gating the active dendritic conductances and burst firing of pyramidal cells through initiation of dendritic spikes (Lovett-Barron et al., 2012; Müller and Remy, 2014). From this perspective, IS3 inhibition of SOM+ cells appears to be crucial in coordination of dendritic inhibition of pyramidal neurons with a direct impact on their input-output conversion and firing behavior.

Under what network conditions might this happen *in vivo?* Based on anatomical data, IS3 cells are likely to be driven by the three major excitatory pathways in the CA1 area: the perforant path, the Schaffer collaterals and the CA1 local collaterals. Additionally, inhibitory input from the CR+ type 1 IS cells may control the activity of IS3 interneurons as CR+ terminals make numerous contacts with CR+ and VIP+ cells (Gulyás et al., 1996). Therefore, the dynamic properties and the relative weight of excitatory and inhibitory inputs converging onto IS3 cells will determine their state-dependent recruitment during ongoing network activity and, accordingly, their role in the recruitment of OLM interneurons *in vivo*. OLM interneurons demonstrate state-dependent fluctuations in activity during network oscillations. In particular, the firing of OLM cells can vary during different episodes or phases of sharp wave ripples (SWRs). For example, in anesthetized animals, OLM cells were quiet during SWRs (Klausberger et al., 2003), whereas in awake, head-fixed animals, OLM cells could fire with a low probability during some SWR episodes (Varga et al., 2012). In freely moving rats, the firing rate of OLM interneurons decreased significantly during sleep compared to awake states and was low during the sleep-associated SWRs (Katona et al., 2014). In addition, OLM cells recorded in slices *in vitro* could fire at a later phase of SWRs (Pangalos et al., 2013). Moreover, both OLM and bistratified cells are strongly modulated during theta oscillations in anesthetized as well as freely-moving animals (Klausberger et al., 2003, 2004; Royer et al., 2012; Varga et al., 2012). In particular, optogenetic experiments revealed that SOM+ dendrite-targeting CA1 interneurons fire at the decay phase of place field during spatial learning, and reduce the firing rate of pyramidal cells without changing the theta phase (Royer et al., 2012). Interestingly, firing of IS3 cells at theta frequency resulted in theta synchronization of OLM cells (Tyan et al., 2014). It is therefore plausible to suggest that IS3 interneurons may increase their firing at specific stages of SWRs and/or theta oscillations *in vivo* and, subsequently, modulate the activity of OLM interneurons.

Recent experimental observations obtained from different neocortical regions highlight the idea that disinhibitory VIP+ interneurons may be engaged in network activity during specific behavioral states (Lee et al., 2013; Pi et al., 2013; Fu et al., 2014). For example, in the somatosensory cortex, the activation of VIP+ interneurons was increased during whisking (Lee et al., 2013). In addition, in the auditory cortex, VIP+ interneurons were strongly recruited by positive and negative reinforcement signals during discrimination tasks (Pi et al., 2013). Furthermore, in the primary sensory cortex, the activity of VIP+ cells was highly increased during locomotion (Fu et al., 2014). Together, these data indicate that VIP+ interneurons may be specialized in controlling the intracortical gating of information during specific behavioral states. Such brain-state-dependent recruitment of VIP+ interneurons points to the important role of the modulatory systems in the regulation of cortical disinhibition. The neuromodulatory effects of dopamine (DA), acetylcholine, and serotonin on pyramidal cells as well as different types of interneurons have been explored in details in different cortical areas. Recent studies have focused on the role of modulators in controlling the recruitment of interneurons in specific behavioral states (Letzkus et al., 2011; Leão et al., 2012; Kimura et al., 2014; Lovett-Barron et al., 2014). For example, it has been reported that dendrite-targeting SOM+ interneurons in the hippocampal CA1 area were recruited by aversive stimuli during contextual fear conditioning through activation of the cholinergic input (Lovett-Barron et al., 2014). VIP+ interneurons in the neocortex express nicotinic acetylcholine receptors (nAChRs; Alitto and Dan, 2013), indicating the potential role of acetylcholine in regulating VIP+ interneuron activity. Indeed, nAChR antagonists strongly attenuated the activation of VIP+ interneurons during behavioral tasks (Fu et al., 2014). Considering the involvement of dopaminergic and cholinergic systems in the reward-associated circuitry (Fukuda et al., 1990; Morris et al., 2004), it is possible that the phasic release of DA and/or acetylcholine, through modulation of VIP+ interneuron activity, may increase their recruitment during reinforcement tasks. Furthermore, in the hippocampus, the dopamine 1 receptor is expressed by CR+ interneurons (Gangarossa et al., 2012). Yet, the role of DA as well as acetylcholine in the recruitment of hippocampal IS3 interneurons remains unexplored.

In conclusion, recent studies from several laboratories provided direct experimental evidence that cortical IS interneurons may play a major role in the state-dependent gating of information flow across cortical regions primarily through dendritic disinhibition of principal neurons. By controlling dendritic electrogenesis and firing mode of principal cells, IS interneurons may determine the functional output of intracortical processing during specific brain states. Future experiments studying the impact of specific connections and their modulation will be required to understand the role of VIP+ interneurons in gating and consolidation of cortical information.

## Conflict of interest statement

The authors declare that the research was conducted in the absence of any commercial or financial relationships that could be construed as a potential conflict of interest.

## References

[B1] Acsády L. Görcs T. J. Freund T. F. (1996). Different populations of vasoactive intestinal polypeptide-immunoreactive interneurons are specialized to control pyramidal cells or interneurons in the hippocampus. Neuroscience 73, 317–334. 10.1016/0306-4522(95)00609-5 8783252

[B2] Alitto H. J. Dan Y. (2013). Cell-type-specific modulation of neocortical activity by basal forebrain input. Front. Syst. Neurosci. 6:79. 10.3389/fnsys.2012.00079 23316142 PMC3540901

[B3] Blasco-Ibáñez J. M. Martínez-Guijarro F. J. Freund T. F. (1998). Enkephalin-containing interneurons are specialized to innervate other interneurons in the hippocampal CA1 region of the rat and guinea-pig. Eur. J. Neurosci. 10, 1784–1795. 10.1046/j.1460-9568.1998.00190.x 9751150

[B4] Caputi A. Rozov A. Blatow M. Monyer H. (2009). Two calretinin-positive GABAergic cell types in layer 2/3 of the mouse neocortex provide different forms of inhibition. Cereb. Cortex 19, 1345–1359. 10.1093/cercor/bhn175 18842664

[B5] Cauli B. Audinat E. Lambolez B. Angulo M. C. Ropert N. Tsuzuki K.. (1997). Molecular and physiological diversity of cortical nonpyramidal cells. J. Neurosci. 17, 3894–3906. 9133407 10.1523/JNEUROSCI.17-10-03894.1997 PMC6573690

[B6] Cauli B. Porter J. T. Tsuzuki K. Lambolez B. Rossier J. Quenet B.. (2000). Classification of fusiform neocortical interneurons based on unsupervised clustering. Proc. Natl. Acad. Sci. U S A 97, 6144–6149. 10.1073/pnas.97.11.6144 10823957 PMC18572

[B7] Cauli B. Zhou X. Tricoire L. Toussay X. Staiger J. F. (2014). Revisiting enigmatic cortical calretinin-expressing interneurons. Front. Neuroanat. 8:52. 10.3389/fnana.2014.00052 25009470 PMC4067953

[B8] Chamberland S. Salesse C. Topolnik D. Topolnik L. (2010). Synapse-specific inhibitory control of hippocampal feedback inhibitory circuit. Front. Cell. Neurosci. 4:130. 10.3389/fncel.2010.00130 21060720 PMC2972748

[B9] Dalezios Y. Luján R. Shigemoto R. Roberts J. D. B. Somogyi P. (2002). Enrichment of mGluR7a in the presynaptic active zones of GABAergic and non-GABAergic terminals on interneurons in the rat somatosensory cortex. Cereb. Cortex 12, 961–974. 10.1093/cercor/12.9.961 12183395

[B10] Dávid C. Schleicher A. Zuschratter W. Staiger J. F. (2007). The innervation of parvalbumin-containing interneurons by VIP-immunopositive interneurons in the primary somatosensory cortex of the adult rat. Eur. J. Neurosci. 25, 2329–2340. 10.1111/j.1460-9568.2007.05496.x 17445231

[B12] Freund T. F. Buzsáki G. (1996). Interneurons of the hippocampus. Hippocampus 6, 347–470. 10.1002/(sici)1098-1063(1996)6:4<347::aid-hipo1>3.0.co;2-i 8915675

[B13] Fu Y. Tucciarone J. M. Espinosa J. S. Sheng N. Darcy D. P. Nicoll R. A.. (2014). A cortical circuit for gain control by behavioral state. Cell 156, 1139–1152. 10.1016/j.cell.2014.01.050 24630718 PMC4041382

[B14] Fukuda M. Ono T. Nakamura K. Tamura R. (1990). Dopamine and ACh involvement in plastic learning by hypothalamic neurons in rats. Brain Res. Bull. 25, 109–114. 10.1016/0361-9230(90)90260-7 2207696

[B15] Galarreta M. Erdélyi F. Szabó G. Hestrin S. (2004). Electrical coupling among irregular-spiking GABAergic interneurons expressing cannabinoid receptors. J. Neurosci. 24, 9770–9778. 10.1523/jneurosci.3027-04.2004 15525762 PMC6730255

[B16] Gangarossa G. Longueville S. De Bundel D. Perroy J. Hervé D. Girault J.-A.. (2012). Characterization of dopamine D1 and D2 receptor-expressing neurons in the mouse hippocampus. Hippocampus 22, 2199–2207. 10.1002/hipo.22044 22777829

[B17] Gonchar Y. Wang Q. Burkhalter A. (2008). Multiple distinct subtypes of GABAergic neurons in mouse visual cortex identified by triple immunostaining. Front. Neuroanat. 1:3. 10.3389/neuro.05.003.2007 18958197 PMC2525923

[B18] Gulyás A. I. Hájos N. Freund T. F. (1996). Interneurons containing calretinin are specialized to control other interneurons in the rat hippocampus. J. Neurosci. 16, 3397–3411. 8627375 10.1523/JNEUROSCI.16-10-03397.1996 PMC6579144

[B19] Katona L. Lapray D. Viney T. J. Oulhaj A. Borhegyi Z. Micklem B. R.. (2014). Sleep and movement differentiates actions of two types of somatostatin-expressing GABAergic interneuron in rat hippocampus. Neuron 82, 872–886. 10.1016/j.neuron.2014.04.007 24794095 PMC4041064

[B20] Kawaguchi Y. Kubota Y. (1997). GABAergic cell subtypes and their synaptic connections in rat frontal cortex. Cereb. Cortex 7, 476–486. 10.1093/cercor/7.6.476 9276173

[B21] Kimura R. Safari M. S. Mirnajafi-Zadeh J. Ebina T. Yanagawa Y. Sohya K.. (2014). Curtailing effect of awakening on visual responses of cortical neurons by cholinergic activation of inhibitory circuits. J. Neurosci. 34, 10122–10133. 10.1523/JNEUROSCI.0863-14.2014 25057213 PMC6608305

[B22] Klausberger T. Magill P. J. Márton L. F. Roberts J. D. Cobden P. M. Buzsáki G.. (2003). Brain-state- and cell-type-specific firing of hippocampal interneurons in vivo. Nature 421, 844–848. 10.1038/nature01374 12594513

[B23] Klausberger T. Márton L. F. Baude A. Roberts J. D. B. Magill P. J. Somogyi P. (2004). Spike timing of dendrite-targeting bistratified cells during hippocampal network oscillations in vivo. Nat. Neurosci. 7, 41–47. 10.1038/nn1159 14634650

[B24] Leão R. N. Mikulovic S. Leão K. E. Munguba H. Gezelius H. Enjin A.. (2012). OLM interneurons differentially modulate CA3 and entorhinal inputs to hippocampal CA1 neurons. Nat. Neurosci. 15, 1524–1530. 10.1038/nn.3235 23042082 PMC3483451

[B25] Lee S. Hjerling-Leffler J. Zagha E. Fishell G. Rudy B. (2010). The largest group of superficial neocortical GABAergic interneurons expresses ionotropic serotonin receptors. J. Neurosci. 30, 16796–16808. 10.1523/JNEUROSCI.1869-10.2010 21159951 PMC3025500

[B26] Lee S. Kruglikov I. Huang Z. J. Fishell G. Rudy B. (2013). A disinhibitory circuit mediates motor integration in the somatosensory cortex. Nat. Neurosci. 16, 1662–1670. 10.1038/nn.3544 24097044 PMC4100076

[B27] Letzkus J. J. Wolff S. B. E. Meyer E. M. M. Tovote P. Courtin J. Herry C.. (2011). A disinhibitory microcircuit for associative fear learning in the auditory cortex. Nature 480, 331–335. 10.1038/nature10674 22158104

[B28] Lovett-Barron M. Kaifosh P. Kheirbek M. A. Danielson N. Zaremba J. D. Reardon T. R.. (2014). Dendritic inhibition in the hippocampus supports fear learning. Science 343, 857–863. 10.1126/science.1247485 24558155 PMC4018419

[B29] Lovett-Barron M. Turi G. F. Kaifosh P. Lee P. H. Bolze F. Sun X.-H.. (2012). Regulation of neuronal input transformations by tunable dendritic inhibition. Nat. Neurosci. 15, 423–430, S1–S3. 10.1038/nn.3024 22246433

[B30] Martina M. Vida I. Jonas P. (2000). Distal initiation and active propagation of action potentials in interneuron dendrites. Science 287, 295–300. 10.1126/science.287.5451.295 10634782

[B31] Miyoshi G. Hjerling-leffler J. Karayannis T. Sousa V. H. Butt J. B. Battiste J.. (2010). Genetic fate mapping reveals that the caudal ganglionic eminence produces a large and diverse population of superficial cortical interneurons. J. Neurosci. 30, 1582–1594. 10.1523/JNEUROSCI.4515-09.2010 20130169 PMC2826846

[B32] Morris G. Arkadir D. Nevet A. Vaadia E. Bergman H. (2004). Coincident but distinct messages of midbrain dopamine and striatal tonically active neurons. Neuron 43, 133–143. 10.1016/j.neuron.2004.06.012 15233923

[B33] Müller C. Remy S. (2014). Dendritic inhibition mediated by O-LM and bistratified interneurons in the hippocampus. Front. Synaptic Neurosci. 6:23. 10.3389/fnsyn.2014.00023 25324774 PMC4179767

[B34] Pangalos M. Donoso J. R. Winterer J. Zivkovic A. R. Kempter R. Maier N.. (2013). Recruitment of oriens-lacunosum-moleculare interneurons during hippocampal ripples. Proc. Natl. Acad. Sci. U S A 110, 4398–4403. 10.1073/pnas.1215496110 23440221 PMC3600450

[B35] Pfeffer C. K. Xue M. He M. Huang Z. J. Scanziani M. (2013). Inhibition of inhibition in visual cortex: the logic of connections between molecularly distinct interneurons. Nat. Neurosci. 16, 1068–1076. 10.1038/nn.3446 23817549 PMC3729586

[B36] Pi H.-J. Hangya B. Kvitsiani D. Sanders J. I. Huang Z. J. Kepecs A. (2013). Cortical interneurons that specialize in disinhibitory control. Nature 503, 521–524. 10.1038/nature12676 24097352 PMC4017628

[B37] Porter J. T. Cauli B. Staiger J. F. Lambolez B. Rossier J. Audinat E. (1998). Properties of bipolar VIPergic interneurons and their excitation by pyramidal neurons in the rat neocortex. Eur. J. Neurosci. 10, 3617–3628. 10.1046/j.1460-9568.1998.00367.x 9875341

[B38] Royer S. Zemelman B. V. Losonczy A. Kim J. Chance F. Magee J. C.. (2012). Control of timing, rate and bursts of hippocampal place cells by dendritic and somatic inhibition. Nat. Neurosci. 15, 769–775. 10.1038/nn.3077 22446878 PMC4919905

[B39] Rudy B. Fishell G. Lee S. Hjerling-Leffler J. (2011). Three groups of interneurons account for nearly 100% of neocortical GABAergic neurons. Dev. Neurobiol. 71, 45–61. 10.1002/dneu.20853 21154909 PMC3556905

[B40] Salesse C. Mueller C. L. Chamberland S. Topolnik L. (2011). Age-dependent remodelling of inhibitory synapses onto hippocampal CA1 oriens-lacunosum moleculare interneurons. J. Physiol. 589, 4885–4901. 10.1113/jphysiol.2011.215244 21825029 PMC3224881

[B41] Staiger J. F. Masanneck C. Schleicher A. Zuschratter W. (2004). Calbindin-containing interneurons are a target for VIP-immunoreactive synapses in rat primary somatosensory cortex. J. Comp. Neurol. 468, 179–189. 10.1002/cne.10953 14648678

[B42] Sugino K. Hempel C. M. Miller M. N. Hattox A. M. Shapiro P. Wu C.. (2006). Molecular taxonomy of major neuronal classes in the adult mouse forebrain. Nat. Neurosci. 9, 99–107. 10.1038/nn1618 16369481

[B43] Tyan L. Chamberland S. Magnin E. Camiré O. Francavilla R. David L. S.. (2014). Dendritic inhibition provided by interneuron-specific cells controls the firing rate and timing of the hippocampal feedback inhibitory circuitry. J. Neurosci. 34, 4534–4547. 10.1523/JNEUROSCI.3813-13.2014 24671999 PMC6608127

[B44] Varga C. Golshani P. Soltesz I. (2012). Frequency-invariant temporal ordering of interneuronal discharges during hippocampal oscillations in awake mice. Proc. Natl. Acad. Sci. U S A 109, E2726–E2734. 10.1073/pnas.1210929109 23010933 PMC3479571

[B45] Xu X. Roby K. D. Callaway E. M. (2010). Immunochemical characterization of inhibitory mouse cortical neurons: three chemically distinct classes of inhibitory cells. J. Comp. Neurol. 518, 389–404. 10.1002/cne.22229 19950390 PMC2804902

